# Differing mental health practice among general practitioners, private psychiatrists and public psychiatrists

**DOI:** 10.1186/1471-2458-5-104

**Published:** 2005-10-07

**Authors:** N Younès, MC Hardy-Bayle, B Falissard, V Kovess, MP Chaillet, I Gasquet

**Affiliations:** 1 Academic Unit of Psychiatry, Centre Hospitalier de Versailles, 177 Rue de Versailles 78157 Le Chesnay Cedex. France; 2 National Institute of Health and Medical Research (INSERM-U669), Hôpital Cochin, AP-HP, Paris, France; 3 Mental Health Foundation, MGEN, Paris, France; 4 Direction of Medical Policy, Assistance Publique – Hôpitaux de Paris, Paris

## Abstract

**Background:**

Providing care for mental health problems concerns General Practitioners (GPs), Private Psychiatrists (PrPs) and Public Psychiatrists (PuPs). As patient distribution and patterns of practice among these professionals are not well known, a survey was planned prior to a re-organisation of mental health services in an area close to Paris

**Methods:**

All GPs (n = 492), PrPs (n = 82) and PuPs (n = 78) in the South-Yvelines area in France were informed of the implementation of a local mental health program. Practitioners interested in taking part were invited to include prospectively all patients with mental health problem they saw over an 8-day period and to complete a 6-month retrospective questionnaire on their mental health practice. 180 GPs (36.6%), 45 PrPs (54.9%) and 63 PuPs (84.0%) responded.

**Results:**

GPs and PrPs were very similar but very different from PuPs for the proportion of patients with anxious or depressive disorders (70% v. 65% v. 38%, p < .001), psychotic disorders (5% v. 7% v. 30%, p < .001), previous psychiatric hospitalization (22% v. 26 v. 61%, p < .001) and receiving disability allowance (16% v. 18% v. 52%, p < .001). GPs had fewer patients with long-standing psychiatric disorders than PrPs and PuPs (52%, 64% v. 63%, p < .001). Time-lapse between consultations was longest for GPs, intermediate for PuPs and shortest for PrPs (36 days v. 26 v. 18, p < .001). Access to care had been delayed longer for Psychiatrists (PrPs, PuPs) than for GPs (61% v. 53% v. 25%, p < .001). GPs and PuPs frequently felt a need for collaboration for their patients, PrPs rarely (42% v. 61%. v. 10%, p < .001).

Satisfaction with mental health practice was low for all categories of physicians (42.6% encountered difficulties hospitalizing patients and 61.4% had patients they would prefer not to cater for). GPs more often reported unsatisfactory relationships with mental health professionals than did PrPs and PuPs (54% v. 15% v. 8%, p < .001).

**Conclusion:**

GP patients with mental health problems are very similar to patients of private psychiatrists; there is a lack of the collaboration felt to be necessary, because of psychiatrists' workload, and because GPs have specific needs in this respect. The "Yvelines-Sud Mental Health Network" has been created to enhance collaboration.

## Background

In developed countries, mental health problems, especially anxious and depressive disorders, are frequent and a leading cause of disability in terms of cost to the individual and society [1-6]. Since they are potentially remediable when adequately treated at an early stage, they represent a major public health challenge [7,8].

Mental Health care concerns the entire health system. First of all, there are general practitioners (GPs) who play a pivotal role, as first line and as the main health professional consulted [1,8-10]. Since primary care is known to be insufficient on its own, access to mental health professionals (psychiatrists, psychologists) needs to be improved to enhance mental health care overall [11-16].

In France up till now patients were free to consult GPs, psychiatrists in private practice in the community (PrPs) or psychiatrists working in the public sector (PuPs). There were 60 815 GPs in France in 1996, and 11 816 PrPs and PuPs in 1997 [17]. Patient distribution, patterns of practice and job satisfaction among these professionals are not well known.

In a pilot area ("Yvelines Sud" area, South-West of Paris), prior to a reorganization of mental health care, a survey was conducted among local physicians involved in mental health care. First General Practitioners' opinions on their practice in mental health and their collaboration with mental health professionals were studied [18]. Then the aim was to gain a better understanding of the overall organization of mental health care. The present article compares general practitioners (GPs), private psychiatrists (PrPs) and public psychiatrists (PuPs) according to their mental health patient population, their mental health practice and their job satisfaction.

## Methods

### Population

The 492 GPs, the 82 PrPs and the 75 PuPs in the area of "South Yvelines" (600 000 inhabitants) were approached by post in spring 2000 and informed of the local mental health program. They were asked if they were willing to recruit for the survey, with a postage-paid reply envelope if they agreed to take part. 180 GPs (response rate of 36.6%), 45 PrPs (54.9%) and 63 PuPs (84.0%) were included. The global response rate is 44.4%.

### Data collected

The mental health professionals responded to two questionnaires requiring approximately 30 minutes to complete:

1. A prospective patient questionnaire completed by the physician.

GPs were asked to include prospectively over an 8-day period all consulting patients over 15 years old for whom a Mental Health Problem was "the main current problem", distinguishing between new patients and those already in follow-up. They were also asked to give the overall number of consultations during the same period. 1519 patients with mental health problems were enrolled by GPs, representing 15.0 % of the overall number of consultations. On average participating GPs saw 8 patients with mental health problems (range 0–35).

PrPs and PuPs were asked to complete the questionnaire in a prospective manner for the first 30 consulting patients, older than 15, also distinguishing new patients from the others. They included 606 new patients and 1645 patients already known to them.

2. A 6-month retrospective practitioner questionnaire measuring the physicians' opinions on their practice in general and on their mental health practice.

### Statistical analyses

Analyses were performed with SAS 8.2 Software. Three groups were considered: GPs, PrPs and PuPs. Descriptive and comparative analyses were carried out on physician demographics, patient profiles, mental health practice and job satisfaction. As appropriate, the chi-square test was used for categorical variables and ANOVA tests for continuous variables. A 5% p level of significance was chosen.

## Results

### Characteristics of respondent physicians (table 1)

**Table 1:** Description of respondent physicians (N = 288)

	General Practitioners N = 180	Private psychiatrists N = 45	Public psychiatrists N = 63
Demographics (%)			
Age class			
25 – 35 years old	9.0	0.0	23.8
36 – 55 years old	84.5	88.9	73.0
56 years old and more	6.2	11.1	3.2
Gender: female	30.6	46.7	37.7
Duration of professional activity			
Less than 5 years	15.0	13.3	47.6
From 5 to 10 years	18.9	28.9	27.0
More than 10 years	66.1	57.8	25.4
			
Professional activity (% of time spent)			
Consultations	76.6	80.2	34.7
Hospitalization	0.0	0.0	31.3
Emergencies	0.0	0.0	8.7
Paper work	10.0	6.3	11.0
Exchanges with colleagues	5.0	3.9	9.9
Further medical education	8.4	9.6	4.4

Respondent physicians were predominantly experienced providers, male and between 36 and 54 years old. PuPs were on average younger than the others (some being residents). Professional activity consisted mostly in clinical activity: consultations for private physicians and more diverse activities for public psychiatrists (also involved in hospitalisation and emergencies). More minor activities were paper work, further education and exchanges with colleagues. 95.6% of PrPs and 65.1% of PuPs reported practising structured psychotherapies (mainly psychoanalysis).

### Comparison of mental health patients of GPs, private and public psychiatrists (table 2)

**Table 2:** Comparison of Patients with mental health problems, already known, seen during one week, for General Practitioners, Private and Public Psychiatrists (N = 2724)

	Patients of General Practitioners N = 1079	Patients of Private Psychiatrists N = 1130	Patients of Public Psychiatrists N = 515	*Chi2 test*
Demographics (%)				
Age class				<0.0001
15 – 25 years old	5.8	9.7	15.7	
26 – 65 years old	78.9	86.9	75.8	
66 years old and more	15.3	3.4	8.5	
Gender: female	68.9	74.7	52.4	<0.0001
Current professional activity	60.1	69.7	44.4	<0.0001
Living alone	25.7	30.0	28.8	ns
Main mental health problem (%)				<0.0001
Anxiety and mood disorders	70.4	65.3	38.4	
Psychotic disorders	4.6	6.9	30.2	
Alcohol and Substance misuse	6.9	1.3	8.0	
Other	18.1	28.3	22.6	
MHP severity (%)				
Past psychiatric hospitalization	21.8	26.1	61.3	<0.0001
National disability allowance	16.2	18.9	51.8	<0.0001
MHP chronicity (%)				
MHP duration more than 3 years	52.2	62.9	63.8	<0.0001

The GP and PrP patients with mental health problems already known to the practitioner were very similar for gender and employment rate. PuP patients were younger, more often male and non-working than GP and PrP patients. There are respectively five times and three times more patients aged 65 or more among GP patients than among PrP and PuP patients.

GPs and PrPs were very similar for percentages of patients diagnosed as anxious or depressed (67.8%) and for percentages of psychotic patients (5.8%). Psychotics patients were much more numerous and anxious or depressed patients much less numerous among PuP patients than among community physician patients. Alcohol and drug misuse were more often treated by GPs and PuPs (7.5%) than by PrPs (1%).

GP and PrP patient percentages did not differ for previous psychiatric hospitalization and national disability allowance. PuPs had patients with more severe characteristics for these variables than GPs and PrPs. Psychiatrists (PrPs and PuPs) had more patients with long term psychiatric disorders than did GPs.

### Comparison of mental health practice between GPs, private and public psychiatrists (table 3)

**Table 3:** Comparison of Mental Health Practice concerning patients seen during one week for General Practitioners, Private and Public Psychiatrists.

	Patients of General Practitioners	Patients of private psychiatrists	Patients of public psychiatrists	*Chi2 test or ANOVA*
New patients (N = 603) (Percentage of MHP patients)	N = 439 (28.9%)	N = 73 (6.1%)	N = 91 (15.1%)	
Patient recruitment (%)				<0.0001
Patient	88.4	61.6	42.8	
Family	14.1	23.3	17.6	
GPs	0.0	26.0	24.2	
Psychiatrist	0.0	6.8	19.8	
Percentage of patients who consulted too late according to the professional (%)	25.2	60.7	52.6	<0.0001
Care project = management by the professional (%)	70.2	89.1	88.6	<0.0001
Wish for collaboration with another physician (%)	42.3	9.6	61.1	<0.0001
				

Patients already known (N= 2724) (Percentage of MHP patients)	N = 1079 (71.1%)	N = 1130 (93.9%)	N = 515 (84.9%)	
Mean days from last consultation (sd)	36.4 (34.9)	17.6 (16.4)	25.7 (18.5)	<0.0001
Collaboration with other professionals (%)	26.3	29.6	53.4	<0.0001
Type of care (%)				
Pharmacological treatment	-	18.9	51.5	
Psychotherapy	-	52.9	16.8	
Both	-	28.2	31.8	

The proportion of new patients among consultants was the highest for GPs, intermediate for PuPs and the lowest for PrPs. Patient recruitment differed: GPs had no patients referred by another physician, while a quarter of psychiatrists' patients were referred by GPs. GPs had fewer new patients for whom they considered that access to mental health care had occurred late. They more often actually sought collaboration for care provision, and much more often stated they would like to have some form of collaboration.

For patients already known to the practitioners, time-lapse between consultations was the longest for GPs, intermediate for PuPs and the shortest for PrPs. Collaboration with another professional less often occurred for community physician patients than for PuP patients. Among psychiatrists, different patterns of care were noted: PrPs were more likely to use psychotherapy than PuPs, and conversely PuPs more often used pharmacological treatments.

### Comparison of job satisfaction of GPs, private and public psychiatrists (table 4)

**Table 4:** Comparison of job satisfaction of General Practitioners, Private and Public Psychiatrists.

	General Practitioners N = 180	Private psychiatrists N = 45	Public psychiatrists N = 63	*Chi2 test*
MHP practice satisfaction (%)				
Having MHP patients that the practitioner would prefer not to cater for	64.2	55.6	64.4	ns
Having difficulties hospitalizing MHP patients (always/often)	46.9	40.0	41.0	ns
Unsatisfactory or very unsatisfactory relationships with				
... GPs	19.0	33.3	29.7	0.05
... private psychiatrists	49.1	11.3	27.1	<0.0001
... public psychiatrists	29.9	29.7	18.8	<0.0001
... colleagues in general	16.2	23.5	10.5	ns
Relationships with mental health professionals are worse than with other health professionals	53.7	15.4	8.1	<0.0001
Having insufficient or very insufficient scope for taking on new patients (workload)	39.7	93.4	77.1	<0.0001
Scope for entrusting part of care to another professional insufficient or very insufficient	46.3	73.2	80.7	<0.0001
				
General practice satisfaction (%)				
- clinical activities				
Independence is essential or important	98.9	100.0	95.2	ns
Exchanges with colleagues are essential or important	99.4	88.8	98.5	ns
Possibility for being replaced insufficient or very insufficient	89.9	86.3	73.3	0.008
- other activities				
Income is unsatisfactory or very unsatisfactory	44.8	56.8	57.7	ns
Administrative duties are demanding or very demanding	91.6	77.8	64.0	<0.0001
Time for further medical education is unsatisfactory or very unsatisfactory	65.7	57.8	77.4	ns
Time for reading medical journals is unsatisfactory or very unsatisfactory	64.4	66.7	78.1	ns
Opportunities for writing medical articles are unsatisfactory or very unsatisfactory	84.1	85.7	80.7	ns
Opportunities for being involved in research and evaluation studies are unsatisfactory or very unsatisfactory	98.3	87.1	68.4	ns

GPs, PrPs and PuPs did not differ according to their general practice satisfaction, except for scope for finding replacements and administrative paperwork: more private physicians (88.1% and 84.7%) complained about these issues than PuPs (73.3% and 64%).

Satisfaction with mental health practice was low for all three categories of physicians: 42.6% encountered difficulties hospitalizing patients and 61.4% had patients they would prefer not to cater for. GPs, PrPs and PuPs however differed according to their mental health practice satisfaction. Psychiatrists (PuPs and especially PrPs) experienced more difficulties in taking on new patients because of workload (88.1%), and in entrusting part of their care to another professional (84.7%) than did GPs. Workload was lower for GPs than for PrPs and PuPs.

Regarding physicians' opinions on their relationships with colleagues, the most frequent unsatisfactory rating was for relationships between GPs and PrPs (for both). The best relationships were among private psychiatrists. GPs more often reported unsatisfactory relationships with mental health professionals than did PrPs and PuPs.

## Discussion

This survey was undertaken to obtain better insight into how practice in mental health is distributed among medical professionals in a French area, prior to re-organisation of mental health services. To our knowledge no other survey has been addressed exhaustively to all physicians involved in mental health care in a particular geographical area. This limits scope for comparisons with other work.

### Limitations

The first limitation is the moderate response rate, reflecting differing interest for the mental health program according to the professional group. GPs may feel less concerned than psychiatrists for different reasons. First, GPs in France, as first line professionals, are contacted by numerous care networks (asthma, diabetes etc) which could take up a lot of their time, even if they are interested in mental health care. Second, GPs may present an interest variable: respondents were probably more involved in mental health care in their ordinary practice than non-respondents. Among psychiatrists, public psychiatrists (PuPs) seemed more concerned than private psychiatrists (PrPs) possibly because they are more concerned about public health issues. Their response rate is comparable to that obtained by studies among Australian or Finnish public psychiatrists [19,20].

The second limitation is that the results are based on reports from the professionals, and particularly in the case of GPs, on their reporting of mental health patients that they themselves identified as having mental health problems. This means of assessment could involve a recruitment bias with a selection of particular patients. However the survey did not intend to assess the prevalence of psychiatric disorders in practice, or needs for mental health treatment, already studied [8,21-24]. The study option was to compare how physicians perceived their usual mental health activity, and how satisfied they were with it, prior to the mental health care reorganization, the aim being to adapt the mental health program to these particular attitudes.

### Mental health patient distribution among professionals

This is the first survey studying mental health patient distribution with a recruitment via the professionals, and comparing GPs, PuPs and PrPs. In particular very few studies have explored PrP practice. In Ontario, Canada, a community survey has shown the influence of certain demographic variables on distribution of patients with mental health problems (age, marital status) but not the influence of severity variables (which were only approximately determined)[21]. In the United States, a large, nationally representative sample of patient visits showed that men, African Americans, other non-white persons, and patients under 15, between 65 and 74, and 75 and over, made proportionally more visits to primary care physicians than to psychiatrists[25]. Severity has been shown to influence the specialist/generalist division of responsibility for patients with mental disorders : specialists were resorted to for patients with psychotic, affective, and schizophrenic disorders, whereas general medical practitioners were more likely treat neurotic disorders in which symptoms of anxiety and depression predominated[24]. Finally in Michigan, USA, a study compared criteria-defined MDD patients of GPs (resorting to primary care) and psychiatrists (outpatients of a university department of psychiatry). Depressed patients consulting a psychiatric practitioner were reported as more severely depressed, more likely to be male, more highly educated and younger. Depressed primary care patients were less likely to have received prior treatment for depression and less likely to present past and current psychiatric comorbidity. The authors concluded that depressed patients encountered in routine primary care are substantially different from those seen in psychiatric settings[22]. The results of the present study confirm the difference between patients with mental health problems encountered in primary care and those encountered in public psychiatric setting (where patients are younger, more often male and more severe). But the difference is smaller between primary care and private psychiatric settings, where patients were in fact more similar than different on demographics, diagnosis and severity criteria. It confirms that GPs had to cater for patients with severe mental health problems. The biggest difference between GP patients and psychiatrist patients was the chronic nature of the mental health problem for the latter, which raises the issues of early help-seeking behaviors in relation to specialist care[26].

### Mental health practice

An important result of the survey lies to the unequal access to mental health care for patients in the light of the first professional consulted: GP, PrP or PuP. Patients with mental health problems seemed fairly similar between primary care and private psychiatric settings. It can be supposed that the first professional consulted is determined by social and educational levels. Whatever the professional category of the practitioner first consulted, these professionals catered for their patients on their own. Thus, the care provided was different. PrPs tended to see their patients more often than did GPs. PrPs were likely to practise psychotherapies while GPs provided other forms of care, without structured psychotherapies.

Regarding mental health practice, PuPs were radically different from both GPs and PrPs: they used more pharmacological treatment and they more often shared practice (team work is more frequent in hospitals); this is coherent with the fact that their particular patients with mental health problems were more frequently psychotic and their condition more severe.

Mental health practice seemed a burden to all professionals (GPs, PrPs and PuPs). Physicians, and especially psychiatrists, were overworked and had difficulty providing the care they considered suitable (hospitalization for instance). This is a problem for all physicians, and not only for PrPs, as shown in Australia where the lack of beds was their most frequent reason for dissatisfaction[19].

The survey showed another aspect that is important for the efficiency of the whole care system: the poor relationships with physicians of other professional categories. GPs, who, as we have shown, manage patients with severe mental health problems but see their patients less often than do PrPs, expressed dissatisfaction with their relationships with psychiatrists. They were particularly dissatisfied with their relationships with PrPs, possibly because they felt closer to them (both are private) so that they may have more expectations in terms of relationships and collaboration with them. GPs desired some form of collaboration for their new patients much more frequently than PrPs. This result evidencing poor relationships among physicians is important because infrequent and unsatisfactory links between primary care and specialist health care are a reason for concern in several countries. It raises the whole issue of help-seeking behaviors [26-29]. However this survey shows that it may be that psychiatrists, overworked and working in isolation, cannot find time or scope for more collaboration with GPs, unless there is a complete reorganization of the mental health system.

The results on job satisfaction among these professionals has revealed a moderate to poor level of satisfaction. All physicians (GPs and psychiatrists) complained about insufficient time for further education and above all, for writing medical articles and for research. Private professionals complained about administrative demands. Time pressure and paperwork have already been shown as frequently reported factors in stress and job dissatisfaction among Australian GPs[30], insufficient participation in research was reported among Canadian psychiatrists[31] and finally, administrative demands were noted among Australian psychiatrists[19]. The present survey did not study litigation and compensation issues, shown to be the most frequent reason for dissatisfaction for private psychiatrists in previous studies in other countries. In France, litigation is still relatively rare. Insufficient time for further education is confirmed by results on time allocation. The main apportionment of waking time is roughly similar when compared with previous studies: first clinical activities then further education and paper work for all professionals, even if this survey did not preclude biased recall of retrospective agendas, as did the survey using a hand-held computer[32]. The present results revealed that physicians without an academic inscription had less time for education and research than other European general physicians in academic departments, American residents or American psychiatrists [32-35].

Finally professionals attached great importance to their clinical independence as well as to scope for collaboration. Physicians' ability to obtain outpatient and inpatient services they required has been shown to be the most consistent and powerful predictor of changes in levels of practice satisfaction over time in an American nationally representative sample of primary care physicians and specialist physicians (including psychiatrists)[36]. This suggests that reorganization of mental health care needs to take account of professionals' dual need for independence and collaboration.

## Conclusion

The present results confirm the need to implement more collaborative practices among practitioners involved in mental health, not in the form of the classic referral to specialists as the major therapeutic option, but in the form of emphasis on collaborative relationships with mental health specialists. Results from this survey have been integrated into the "South Yvelines Mental Health Network" created in June 2001, by promoting this type of collaborative relationships in the area (workshops, educational interventions, targeted collaborative actions ...). It was organized along the lines of the "individualized stepped care" proposed by Von Korff and colleagues[37,38]. For example, patients who pose problem for their primary care physician will benefit from prompt public psychiatric consultations, or brief interventions in support of primary care management without transferring the responsibility to specialist care. Only if necessary, will the transfer to specialist care by private or publics psychiatrists be organized. Further evaluations of the impact of the South Yvelines Mental Health Network are in completion.

## List of abbreviations

GPs (General Practitioners). PrPs (Private Psychiatrists). PuPs (Public Psychiatrists).

## Competing interests

The author(s) declare that they have no competing interests.

## Authors' contributions

Study concept and design: Gasquet, Kovess, Hardy-Bayle. Acquisition of data, study supervision : Chaillet. Analysis and interpretation: Younès, Gasquet. Drafting of the manuscript : Younès. Statistical expertise : Younès, Falissard. Critical revision : Gasquet, Younès, Falissard, Hardy-Bayle

## Pre-publication history

The pre-publication history for this paper can be accessed here:



## Supplementary Material

Additional File 1 Mental Health Practice Questionnaire. The questionnaire is divided into two parts : a retrospective questionnaire on professional activity and a prospective patient questionnaire. Click here for file

Additional File 2 Questionnaire sur les Pratiques en Santé Mentale. French version of the Mental Health Practice Questionnaire. Click here for file
