# Supplementary material for: Differing mental health practice among general practitioners, private psychiatrists and public psychiatrists
Source: BMC Public Health. 2005 Oct 7;5:104. doi: 10.1186/1471-2458-5-104 (PMC1266376; doi:10.1186/1471-2458-5-104)
Supplement: Additional File 1 — Mental Health Practice Questionnaire. The questionnaire is divided into two parts : a retrospective questionnaire on professional activity and a prospective patient questionnaire. [file 1471-2458-5-104-S1.doc]

**mental health practice questionnaire**

*IMPORTANT NOTE : The following questionnaire is an English translation of the questionnaire used for data collection for the study, drafted by an English native speaker with experience in questionnaire translation and adaptation. In this form it is designed to provide information on the content of the French questionnaire used in the study, and may not be suitable without adaptation for use in another cultural and therapeutic context.*

This questionnaire, which requires approximately 30 minutes to complete, is divided into two parts :

1. a retrospective questionnaire on your professional activity over the last 6 months
2. a prospective patient questionnaire

Patient inclusion: inclusion of patients aged **15 or more** over a period of one week.

GPs: recruit all consulting patients for whom a Mental Health Problem is the main current problem (**new** patient or **already in follow-up**) within the chosen week.

Psychiatrists: recruit the first 30 **new** patients **and** the first 30 patients **already in follow-up** consulting in the course of the chosen week..

**practitioner questionnaire**

| Your age group | 25 - 35 years old |  |  |
| --- | --- | --- | --- |
|  | 36 - 55 years old |  |  |
|  | 56 years old and more |  |  |
|  |  |  |  |
| Gender | Male |  |  |
|  | Female |  |  |
|  |  |  |  |
| Years of professional activity | Less than 5 years |  |  |
|  | From 5 to 10 years |  |  |
|  | More than 10 years |  |  |

**Professional activity**

*NB: these questions refer to the last 6 months*

- In your professional activity, indicate the percentage of time spent on each of those activities

| Consultations |  | % |
| --- | --- | --- |
| Hospitalisations |  | % |
| Emergencies |  | % |
| Paper work |  | % |
| Further medical education |  | % |
| Exchanges with colleagues for patient care |  | % |

- Do you practise structured psychotherapies ?

| No |  |  |  |  |  |
| --- | --- | --- | --- | --- | --- |
| Yes |  |  | → of what type? | Cognitive-behavioural |  |
|  |  |  |  | Psychoanalysis |  |
|  |  |  |  | Family therapy |  |
|  |  |  |  | Other |  |

*Please respond to the statements below using the response choices provided*

- The income I earn is…

| Very satisfactory |  |  |
| --- | --- | --- |
| Fairly satisfactory |  |  |
| Rather unsatisfactory |  |  |
| Very unsatisfactory |  |  |
| No opinion |  |  |

- Administrative duties are demanding or very demanding.

| Fully agree |  |  |
| --- | --- | --- |
| Rather agree |  |  |
| Rather disagree |  |  |
| Completely disagree |  |  |
| No opinion |  |  |

- Scope for finding replacements is insufficient.

| Fully agree |  |  |
| --- | --- | --- |
| Rather agree |  |  |
| Rather disagree |  |  |
| Completely disagree |  |  |
| No opinion |  |  |

- Time available for further medical education is …

| Very satisfactory |  |  |
| --- | --- | --- |
| Fairly satisfactory |  |  |
| Rather unsatisfactory |  |  |
| Very unsatisfactory |  |  |
| No opinion |  |  |

- Time available for reading medical journals is …

| Very satisfactory |  |  |
| --- | --- | --- |
| Fairly satisfactory |  |  |
| Rather unsatisfactory |  |  |
| Very unsatisfactory |  |  |
| No opinion |  |  |

- Time available for writing medical articles is…

| Very satisfactory |  |  |
| --- | --- | --- |
| Fairly satisfactory |  |  |
| Rather unsatisfactory |  |  |
| Very unsatisfactory |  |  |
| No opinion |  |  |

- Opportunities for being involved in research and evaluation studies are…

| Very satisfactory |  |  |
| --- | --- | --- |
| Fairly satisfactory |  |  |
| Rather unsatisfactory |  |  |
| Very unsatisfactory |  |  |
| No opinion |  |  |

- How important do you consider your professional independence to be ?

| Essential |  |  | Important |  |  | Not very important |  |  | Not at all important |  |  |
| --- | --- | --- | --- | --- | --- | --- | --- | --- | --- | --- | --- |

- How important do you consider exchanges with colleagues to be ?

| Essential |  |  | Important |  |  | Not very important |  |  | Not at all important |  |  |
| --- | --- | --- | --- | --- | --- | --- | --- | --- | --- | --- | --- |

**mental health practice**

- It is difficult for me to take on new patients with mental health problems (e.g. because of workload)

| Fully agree |  |  |
| --- | --- | --- |
| Rather agree |  |  |
| Rather disagree |  |  |
| Completely disagree |  |  |
| No opinion |  |  |

- I have difficulties hospitalising patients with mental health problems.

| Very often or always |  |  |
| --- | --- | --- |
| Fairly Often |  |  |
| Not very often |  |  |
| Very rarely or never |  |  |
| No opinion |  |  |

- Scope for entrusting part of patient care to another professional is inadequate.

| Fully agree |  |  |
| --- | --- | --- |
| Rather agree |  |  |
| Rather disagree |  |  |
| Completely disagree |  |  |
| No opinion |  |  |

- Do you have patients with mental health problems that you would prefer not to cater for ?

| Yes |  |  |
| --- | --- | --- |
| No |  |  |

- The quality of relationships with general practitioners colleagues is …

| Very satisfactory |  |  |
| --- | --- | --- |
| Fairly satisfactory |  |  |
| Rather unsatisfactory |  |  |
| Very unsatisfactory |  |  |
| No opinion |  |  |

- Quality of relationships with private psychiatrist colleagues is …

| Very satisfactory |  |  |
| --- | --- | --- |
| Fairly satisfactory |  |  |
| Rather unsatisfactory |  |  |
| Very unsatisfactory |  |  |
| No opinion |  |  |

- Quality of relationships with public psychiatrist colleagues is …

| Very satisfactory |  |  |
| --- | --- | --- |
| Fairly satisfactory |  |  |
| Fairly unsatisfactory |  |  |
| Very unsatisfactory |  |  |
| No opinion |  |  |

- How do you evaluate your relationships with mental health professionals compared to relationships with other health professionals ?

| Much better |  |  |
| --- | --- | --- |
| Better |  |  |
| Same |  |  |
| Worse |  |  |
| No opinion |  |  |

**patient questionnaire**

**NEW PATIENT**

demographics

| Age group | 15 - 25 years old |  |  |
| --- | --- | --- | --- |
|  | 26 - 65 years old |  |  |
|  | 66 years old and more |  |  |
|  |  |  |  |
| Gender | Male |  |  |
|  | Female |  |  |
|  |  |  |  |
| Current professional activity | Yes |  |  |
|  | No |  |  |
|  |  |  |  |
| Does he/she live alone? | Yes |  |  |
|  | No |  |  |

**mental health problem**

- Who, if anyone, referred the patient?

| Patient consulted spontaneously |  |  |
| --- | --- | --- |
| Family |  | % |
| Psychiatrist |  | % |
| GP |  |  |
| Other |  |  |

- How long has the mental health problem lasted ?

| < 1 year |  |  |
| --- | --- | --- |
| 1-3 years |  |  |
| > 3 years |  |  |

- Has there been psychiatric hospitalisation in the past?

| Yes |  |  |
| --- | --- | --- |
| No |  |  |
| No information |  |  |

- Does the patient have a national disability allowance for psychiatric reasons?

| Yes |  |  |
| --- | --- | --- |
| No |  |  |
| No information |  |  |

- What is your diagnosis for the mental health problem ?

| Anxiety disorders |  |  |
| --- | --- | --- |
| Mood disorders |  |  |
| Psychotic disorders |  |  |
| Alcohol and Substance misuse |  |  |
| Suicide attempt or suicidal ideas |  |  |
| Eating disorders |  |  |
| Other |  |  |
| Please specify...................................... | | |

- Do you think that this patient should have consulted a mental health specialist earlier?

| Yes |  |  |
| --- | --- | --- |
| No |  |  |
| No opinion |  |  |

- Do you think that follow-up is required for this patient ?

| Yes |  |  |
| --- | --- | --- |
| No |  |  |

- Will you see the patient again?

| Yes |  |  | → Time until the next appointment (days) |  |  |  |
| --- | --- | --- | --- | --- | --- | --- |
| No |  |  |  |  |  |  |

- Would you like to have the collaboration of another physician for this patient’s care?

| No |  |  |  |  |  |
| --- | --- | --- | --- | --- | --- |
| Yes |  |  | → Which type ? | Private psychiatrist |  |
|  |  |  |  | Public psychiatrist |  |
|  |  |  |  | Psychologist |  |
|  |  |  |  | Other |  |

- Did you refer the patient to another physician?

| No |  |  |  |  |  |
| --- | --- | --- | --- | --- | --- |
| Yes |  |  | → To whom? | Private psychiatrist |  |
|  |  |  |  | Public psychiatrist |  |
|  |  |  |  | Psychologist |  |
|  |  |  |  | Other |  |

- Duration of consultation (minutes)

|  |  |  |
| --- | --- | --- |

**Patient already in follow-up**

demographics

| Age group | 15 - 25 years old |  |  |
| --- | --- | --- | --- |
|  | 26 - 65 years old |  |  |
|  | 66 years old and more |  |  |
|  |  |  |  |
| Gender | Male |  |  |
|  | Female |  |  |
|  |  |  |  |
| Current professional activity | Yes |  |  |
|  | No |  |  |
|  |  |  |  |
| Does he/she live alone? | Yes |  |  |
|  | No |  |  |

**mental health problem**

- How long has the mental health problem lasted ?

| < 1 year |  |  |
| --- | --- | --- |
| 1-3 years |  |  |
| > 3 years |  |  |

- Has there been psychiatric hospitalisation in the past?

| Yes |  |  |
| --- | --- | --- |
| No |  |  |
| No information |  |  |

- Does the patient have a national disability allowance for psychiatric reasons?

| Yes |  |  |
| --- | --- | --- |
| No |  |  |
| No information |  |  |

- What is your diagnosis for the mental health problem ?

| Anxiety disorders |  |  |
| --- | --- | --- |
| Mood disorders |  |  |
| Psychotic disorders |  |  |
| Alcohol and Substance misuse |  |  |
| Suicide attempt or suicidal ideas |  |  |
| Eating disorders |  |  |
| Other |  |  |
| Please specify...................................... | | |

- What type of care are you providing for this patient?

| Pharmacological treatment |  |  |
| --- | --- | --- |
| Psychotherapy |  |  |
| Both |  |  |

- Time lapse since the last consultation (days)

|  |  |  |
| --- | --- | --- |

- Do you have the collaboration of another physician for the care of this patient?

| No |  |  |  |  |  |
| --- | --- | --- | --- | --- | --- |
| Yes |  |  | → What type ? | Private psychiatrist |  |
|  |  |  |  | Public psychiatrist |  |
|  |  |  |  | Psychologist |  |
|  |  |  |  | Other |  |
