# Supplementary material for: Differing mental health practice among general practitioners, private psychiatrists and public psychiatrists
Source: BMC Public Health. 2005 Oct 7;5:104. doi: 10.1186/1471-2458-5-104 (PMC1266376; doi:10.1186/1471-2458-5-104)
Supplement: Additional File 2 — Questionnaire sur les Pratiques en Santé Mentale. French version of the Mental Health Practice Questionnaire. [file 1471-2458-5-104-S2.doc]

**questionnaire de pratiques en sante mentale**

Ce questionnaire nécessite environ 30 minutes pour être complété et comprend deux parties :

1. Un questionnaire rétrospectif de l’activité professionnelle sur les 6 derniers mois
2. Un questionnaire patient prospectif

Inclusion de tous les patients âgés de plus de 15 ans sur une semaine donnée.

Médecin Généraliste: recruter tous les patients vus en consultation pendant la semaine pour lesquels un problème psychologique est dominant (en distinguant les nouveaux patients et les patients en cours de suivis).

Psychiatres: recruter les 30 premiers nouveaux patients et les 30 premiers patients déjà suivis vus en consultation pendant la semaine.

**questionnaire “praticien”**

| Groupe d’âge | 25 - 35 ans |  |  |
| --- | --- | --- | --- |
|  | 36 - 55 ans |  |  |
|  | 56 ans et plus |  |  |
|  |  |  |  |
| Sexe | Homme |  |  |
|  | Femme |  |  |
|  |  |  |  |
| Ancienneté d’activité | Moins de 5 ans |  |  |
|  | De 5 à 10 ans |  |  |
|  | Plus de 10 ans |  |  |

**activite professionnelle**

*NB :ces questions concernent votre activité au cours des 6 derniers mois*

- Dans votre activité professionnelle, précisez le pourcentage de temps consacré à chacune de ces activités

| Consultations |  | % |
| --- | --- | --- |
| Hospitalisations |  | % |
| Urgences |  | % |
| Tâches administratives |  | % |
| Formation |  | % |
| Echanges avec les partenaires de soins pour le suivi des patients |  | % |

- Pratiquez-vous des psychothérapies structurées ?

| Non |  |  |  |  |  |
| --- | --- | --- | --- | --- | --- |
| Oui |  |  | → de quel type? | Cognitivo-comportementale |  |
|  |  |  |  | Psychanalytiques |  |
|  |  |  |  | Systémique |  |
|  |  |  |  | Autres |  |

*Choisissez une réponse pour chaque proposition (par rapport à ce que vous souhaiteriez dans l’idéal)*

- Les revenus liés à mon activité sont…

| Très satisfaisants |  |  |
| --- | --- | --- |
| Satisfaisants |  |  |
| Insatisfaisants |  |  |
| Très insatisfaisants |  |  |
| Sans avis |  |  |

- Les charges administratives sont lourdes.

| Tout à fait vrai |  |  |
| --- | --- | --- |
| Plutôt vrai |  |  |
| Plutôt faux |  |  |
| Tout à fait faux |  |  |
| Sans avis |  |  |

- Les possibilités de remplacement sont insuffisantes.

| Tout à fait vrai |  |  |
| --- | --- | --- |
| Plutôt vrai |  |  |
| Plutôt faux |  |  |
| Tout à fait faux |  |  |
| Sans avis |  |  |

- Le temps consacré à ma formation médicale continue est…

| Très satisfaisant |  |  |
| --- | --- | --- |
| Satisfaisant |  |  |
| Insatisfaisant |  |  |
| Très insatisfaisant |  |  |
| Sans avis |  |  |

- Le temps que je peux passer à lire des journaux médicaux est…

| Très satisfaisant |  |  |
| --- | --- | --- |
| Satisfaisant |  |  |
| Insatisfaisant |  |  |
| Très insatisfaisant |  |  |
| Sans avis |  |  |

- Le temps que je peux passer à écrire des articles médicaux est…

| Très satisfaisant |  |  |
| --- | --- | --- |
| Satisfaisant |  |  |
| Insatisfaisant |  |  |
| Très insatisfaisant |  |  |
| Sans avis |  |  |

- Mes possibilités de participer à des travaux de recherche et à l’évaluation des pratiques sont…

| Très satisfaisantes |  |  |
| --- | --- | --- |
| Satisfaisantes |  |  |
| Insatisfaisantes |  |  |
| Très insatisfaisantes |  |  |
| Sans avis |  |  |

- Quelle importance attribuez-vous à votre autonomie professionnelle ?

| Essentielle |  |  | Importante |  |  | Peu importante |  |  | Sans importance |  |  |
| --- | --- | --- | --- | --- | --- | --- | --- | --- | --- | --- | --- |

- Quelle importance attribuez-vous à vos échanges avec vos collègues ?

| Essentiels |  |  | Importants |  |  | Peu importants |  |  | Sans importance |  |  |
| --- | --- | --- | --- | --- | --- | --- | --- | --- | --- | --- | --- |

**pratique en sante mentale**

- J’ai des difficultés à assurer les nouvelles demandes de patients (à cause de la saturation)

| Tout à fait vrai |  |  |
| --- | --- | --- |
| Plutôt vrai |  |  |
| Plutôt faux |  |  |
| Tout à fait faux |  |  |
| Sans avis |  |  |

- J’ai des difficultés pour faire hospitaliser les patients avec des problèmes mentaux.

| Très souvent ou toujours |  |  |
| --- | --- | --- |
| Souvent |  |  |
| Rarement |  |  |
| Très rarement ou jamais |  |  |
| Sans avis |  |  |

- Les possibilités de confier certaines pratiques à d’autres collègues sont insuffisantes.

| Tout à fait vrai |  |  |
| --- | --- | --- |
| Plutôt vrai |  |  |
| Plutôt faux |  |  |
| Tout à fait faux |  |  |
| Sans avis |  |  |

- Prenez-vous en charge des patients que vous souhaiteriez ne pas suivre?

| Oui |  |  |
| --- | --- | --- |
| Non |  |  |

- La qualité des échanges avec mes collègues médecins généralistes est…

| Très satisfaisante |  |  |
| --- | --- | --- |
| Satisfaisante |  |  |
| Insatisfaisante |  |  |
| Très insatisfaisante |  |  |
| Sans avis |  |  |

- La qualité des échanges avec mes collègues psychiatres privés est…

| Très satisfaisante |  |  |
| --- | --- | --- |
| Satisfaisante |  |  |
| Insatisfaisante |  |  |
| Très insatisfaisante |  |  |
| Sans avis |  |  |

- La qualité des échanges avec mes collègues psychiatres publics est…

| Très satisfaisante |  |  |
| --- | --- | --- |
| Satisfaisante |  |  |
| Insatisfaisante |  |  |
| Très insatisfaisante |  |  |
| Sans avis |  |  |

- Comment jugez-vous vos échanges avec les professionnels de santé mentale par rapport aux échanges avec les professionels hors santé mentale ?

| Bien meilleur |  |  |
| --- | --- | --- |
| Meilleur |  |  |
| Identique |  |  |
| Moins bon |  |  |
| Sans avis |  |  |

**questionnaire “patient”**

**Nouveaux patients**

demographie

| Groupe d’âge | 15 - 25 ans |  |  |
| --- | --- | --- | --- |
|  | 26 - 65 ans |  |  |
|  | 66 ans et plus |  |  |
|  |  |  |  |
| Sexe | Homme |  |  |
|  | Femme |  |  |
|  |  |  |  |
| Activité professionnelle actuelle | Oui |  |  |
|  | Non |  |  |
|  |  |  |  |
| Vit-il (elle) seul(e)? | Oui |  |  |
|  | Non |  |  |

**probleme mental**

- Qui a adressé le patient?

| Le patient lui-même |  |  |
| --- | --- | --- |
| La famille |  | % |
| Un psychiatre |  | % |
| Un médecin généraliste |  |  |
| Autres |  |  |

- Quelle est l’ancienneté du problème psychiatrique ?

| < 1 an |  |  |
| --- | --- | --- |
| 1-3 ans |  |  |
| > 3 ans |  |  |

- Y a-t-il eu hospitalisation antérieure pour le motif psychiatrique?

| Oui |  |  |
| --- | --- | --- |
| Non |  |  |
| Sans information |  |  |

- Le patient bénéficie-t-il d’une Allocation adulte handicapé ou d’une ALD ou invalidité pour motif psychiatrique?

| Oui |  |  |
| --- | --- | --- |
| Non |  |  |
| Sans information |  |  |

- Quel diagnostic portez-vous pour le problème mental ?

| Trouble anxieux |  |  |
| --- | --- | --- |
| Trouble de l’humeur |  |  |
| Trouble psychotique |  |  |
| Abus d’alcool ou de substances |  |  |
| Tentative de suicide ou crise suicidaire |  |  |
| Troubles des conduites alimentaires |  |  |
| Autres |  |  |
| Spécifier...................................... | | |

- Selon vous, le patient que vous voyez aurait-il bénéficié d’une prise en charge plus précoce par un spécialiste en santé mentale?

| Oui |  |  |
| --- | --- | --- |
| Non |  |  |
| Sans avis |  |  |

- Pensez-vous qu’un suivi soit nécessaire ?

| Oui |  |  |
| --- | --- | --- |
| Non |  |  |

- Allez-vous revoir le patient?

| Oui |  |  | → Délai de la prochaine consultation (jours) |  |  |  |
| --- | --- | --- | --- | --- | --- | --- |
| Non |  |  |  |  |  |  |

- Souhaiteriez-vous faire intervenir d’autres professionnels dans le suivi psychiatrique de ce patient?

| Non |  |  |  |  |  |
| --- | --- | --- | --- | --- | --- |
| Oui |  |  | → Qui ? | Un psychiatre libéral |  |
|  |  |  |  | Un psychiatre public |  |
|  |  |  |  | Un psychologue |  |
|  |  |  |  | Autres |  |

- Adressez-vous le patient à un autre professionnel?

| Non |  |  |  |  |  |
| --- | --- | --- | --- | --- | --- |
| Oui |  |  | → A qui ? | Un psychiatre libéral |  |
|  |  |  |  | Un psychiatre public |  |
|  |  |  |  | Un psychologue |  |
|  |  |  |  | Autres |  |

- Durée de la consultation (minutes)

|  |  |  |
| --- | --- | --- |

**patients en cours de suivi**

demographie

| Groupe d’âge | 15 - 25 ans |  |  |
| --- | --- | --- | --- |
|  | 26 - 65 ans |  |  |
|  | 66 ans et plus |  |  |
|  |  |  |  |
| Sexe | Homme |  |  |
|  | Femme |  |  |
|  |  |  |  |
| Activité professionnelle actuelle | Oui |  |  |
|  | Non |  |  |
|  |  |  |  |
| Vit-il (elle) seul(e)? | Oui |  |  |
|  | Non |  |  |

**probleme mental**

- Quelle est l’ancienneté du problème psychiatrique ?

| < 1 an |  |  |
| --- | --- | --- |
| 1-3 ans |  |  |
| > 3 ans |  |  |

- Y a-t-il eu hospitalisation antérieure pour le motif psychiatrique?

| Oui |  |  |
| --- | --- | --- |
| Non |  |  |
| Sans information |  |  |

- Le patient bénéficie-t-il d’une Allocation adulte handicapé ou d’une ALD ou invalidité pour motif psychiatrique?

| Oui |  |  |
| --- | --- | --- |
| Non |  |  |
| Sans information |  |  |

- Quel diagnostic portez-vous pour le problème mental ?

| Trouble anxieux |  |  |
| --- | --- | --- |
| Trouble de l’humeur |  |  |
| Trouble psychotique |  |  |
| Abus d’alcool ou de substances |  |  |
| Tentative de suicide ou crise suicidaire |  |  |
| Troubles des conduites alimentaires |  |  |
| Autres |  |  |
| Specifier...................................... | | |

- Quel type de prise en charge prodiguez-vous pour ce patient?

| Traitement Pharmacologique |  |  |
| --- | --- | --- |
| Psychotherapie |  |  |
| Les deux |  |  |

- Délai depuis la dernière consultation (jours)

|  |  |  |
| --- | --- | --- |

- D’autres professionnels interviennent-ils dans le suivi psychiatrique de ce patient?

| Non |  |  |  |  |  |
| --- | --- | --- | --- | --- | --- |
| Oui |  |  | → Qui ? | Un psychiatre libéral |  |
|  |  |  |  | Un psychiatre public |  |
|  |  |  |  | Un psychologue |  |
|  |  |  |  | Autres |  |
